# Otopathogens Detected in Middle Ear Fluid Obtained during Tympanostomy Tube Insertion: Contrasting Purulent and Non-Purulent Effusions

**DOI:** 10.1371/journal.pone.0128606

**Published:** 2015-06-03

**Authors:** Robert C. Holder, Daniel J. Kirse, Adele K. Evans, Amy S. Whigham, Timothy R. Peters, Katherine A. Poehling, William E. Swords, Sean D. Reid

**Affiliations:** 1 Department of Microbiology and Immunology, Wake Forest School of Medicine, Winston-Salem, North Carolina, United States of America; 2 Department of Otolaryngology-Head and Neck Surgery, Wake Forest School of Medicine, Winston-Salem, North Carolina, United States of America; 3 Department of Pediatrics, Wake Forest School of Medicine, Winston-Salem, North Carolina, United States of America; 4 Department of Epidemiology and Prevention, Wake Forest School of Medicine, Winston-Salem, North Carolina, United States of America; 5 Department of Microbiology and Immunology, Ross University School of Medicine, Roseau, Dominica, West Indies; Centers for Disease Control & Prevention, UNITED STATES

## Abstract

Otitis media is a prominent disease among children. Previous literature indicates that otitis media is a polymicrobial disease, with *Haemophilus influenzae*, *Streptococcus pneumoniae*, *Alloiococcus otitidis* and *Moraxella catarrhalis* being the most commonly associated bacterial pathogens. Recent literature suggests that introduction of pneumococcal conjugate vaccines has had an effect on the etiology of otitis media. Using a multiplex PCR procedure, we sought to investigate the presence of the aforementioned bacterial pathogens in middle ear fluid collected from children undergoing routine tympanostomy tube placement at Wake Forest Baptist Medical Center during the period between January 2011 and March 2014. In purulent effusions, one or more bacterial organisms were detected in ~90% of samples. Most often the presence of *H*. *influenzae* alone was detected in purulent effusions (32%; 10 of 31). In non-purulent effusions, the most prevalent organism detected was *A*. *otitidis* (26%; 63 of 245). Half of the non-purulent effusions had none of these otopathogens detected. In purulent and non-purulent effusions, the overall presence of *S*. *pneumoniae* was lower (19%; 6 of 31, and 4%; 9 of 245, respectively) than that of the other pathogens being identified. The ratio of the percentage of each otopathogen identified in purulent vs. non-purulent effusions was >1 for the classic otopathogens but not for *A*. *otitidis*.

## Introduction

Otitis media (OM) is a leading cause for outpatient visits as well as antibiotic prescriptions for children[1–5]. It is estimated that by 3 years of age 80% of children will have experienced at least one case of OM[6, 7], and 40% of these children will experience six or more recurrences by 7 years of age[8]. Annual direct costs associated with OM are estimated to approach $3–5 billion[9–11], and indirect costs which include loss of productivity and lost working days by family members drive this value higher[12, 13].

Distinct from our previous work[14], this study focuses on the prevalence of four otopathogens (*Haemophilus influenzae*, *Streptococcus pneumoniae*, *Alloiococcus otitidis*, and *Moraxella catarrhalis*) in middle ear fluid collected from children undergoing tympanostomy tube placement at Wake Forest Baptist Medical Center (Winston-Salem, NC, USA) from January 2011 until March 2014, after the introduction of the pneumococcal conjugate vaccine PCV13.

## Materials and Methods

### Subjects and Sample Collection

Children who had tympanostomy tube placement at Wake Forest Baptist Medical Center from January 2011 through March 2014 and had middle ear fluid at the time of surgery were eligible. Discarded samples of middle ear effusions were evaluated for bacterial pathogens, as described below.

Study specimens were collected from middle ears that had effusions at the time of tympanostomy tube placement. Fluid was aspirated into a sterile trap. Specimens were categorized as purulent or non-purulent fluid behind the tympanic membrane by the otolaryngologist at the time of tympanostomy tube placement. Fluids visually observed by the pediatric otolaryngologist to have a whitish appearance with a milky or mucoid consistency were designated as purulent. All middle ear effusion samples were kept at room temperature and transported to the research laboratory within 2 hours and then refrigerated at 4°C until DNA isolation. Samples were processed within 5 days of refrigeration.

### Isolation of DNA

DNA was extracted from middle ear effusions as described previously[14]. Briefly, middle ear effusions were incubated with various degradation components (mutanolysin, lysozyme, sodium dodecyl sulfate (SDS), RNase, proteinase K) to disrupt bacterial cell walls, remove RNA, and degrade proteins. Remaining DNA was phenol:chloroform:isoamyl alcohol extracted and ethanol precipitated. Resuspended DNA precipitates were used as template material in the multiplex PCR procedure.

### Polymerase Chain Reaction

A multiplex PCR procedure created by Hendolin et al[15] was used with minor modification to simultaneously detect *H*. *influenzae*, *S*. *pneumoniae*, *A*. *otitidis*, and *M*. *catarrhalis* in middle ear effusions, as previously described[14]. The modification mentioned above was that the polymerase used in the procedure (5 PRIME HotMasterMix) required an extension temperature of 65°C rather than the often used 72°C associated with Taq polymerase.

### Chart Review

This study was reviewed and approved by the Wake Forest School of Medicine Institutional Review Board. Sex, age, medications, clinical characteristics and prior antimicrobial treatments were collected from the subject record without identifiable information. Although identifiable information was not collected, administrative data reveals that the average age of children who had tympanostomy tubes placed during this study period was 2.7 years (range of 0–18 years). The demographic characteristics, current medications, previous antibiotic use, and past medical history were all determined by medical record review using a standardized clinical form. The medical record typically contained notes about ear infections from the primary care provider as well as clinic visits to the otolaryngologist.

### Analyses

We compared the demographic characteristics, clinical characteristics, or microbiologic results of children with purulent and non-purulent middle ear effusions by using chi-square analyses or Fisher's exact tests. For children with bilateral middle ear effusions, the results from each ear were combined so that each child had one result. The unadjusted odds ratio and 95% confidence intervals were calculated for each variable. A multivariate logistic regression analysis was performed to compute the odds of a purulent infection as compared to a non-purulent infection for each of four otopathogens and age groups. We lacked sufficient power to analyze other variables. STATA 12.1 (College Station, TX) was used for all statistical analyses.

## Results

**Fig 1:**
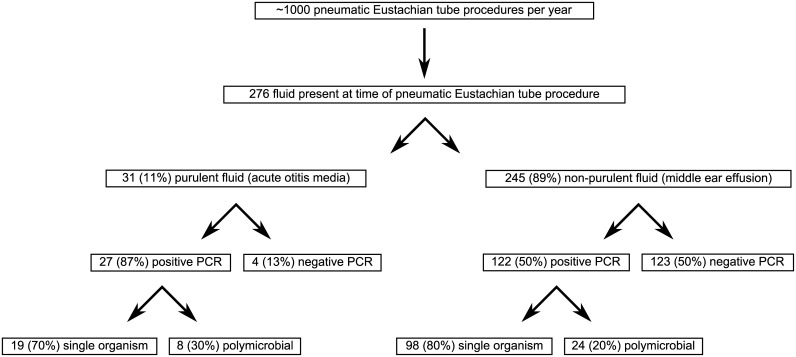
Flow Chart of PCR Analysis.

A total of 276 children had middle ear fluid collected at the time of tympanostomy tube placement from January 2011 through March 2014 (Fig 1). Half were 1–3 years of age, two-thirds were male, and 58% were White (Table 1). Children with purulent effusions were more likely to be 1–3 years of age, to have a history of ear infections, to have been on antibiotics within the past 6 months, and to be on antibiotics at the time of surgery than children with non-purulent effusions.

**Table 1:** Subject Demographics.

		Total (n = 276)	Purulent Effusions (n = 31)	Non-purulent Effusions (n = 245)	
		No. of Children (Column %*)	No. of Children (Column %*)	No. of Children (Column %*)	Unadjusted Odds Ratio (95% Confidence Interval)
Age	<1 year	32 (12)	8 (26)	24 (10)	Reference
	1–3 years	136 (49)	20 (65)	116 (47)	0.5 (0.2–1.3)
	>3 years	108 (39)	3 (10)	105 (43)	0.09 (0.02–0.35)
Gender	Female	94 (34)	11 (35)	83 (34)	Reference
	Male	182 (66)	20 (65)	162 (66)	0.9 (0.4–2.0)
Race	White	158 (58)	24 (77)	134 (56)	Reference
	Black	65 (24)	5 (16)	60 (25)	0.47 (0.17–1.28)
	Hispanic/Other	49 (18)	2 (6)	47 (20)	0.24 (0.05–1.04)
Currently** on Antibiotics^a^	No	245 (89)	23 (74)	222 (91)	Reference
	Yes	31 (11)	8 (26)	23 (9)	3.4 (1.3–8.4)
Previously on Antibiotics^b^	No	144 (52)	6 (19)	138 (56)	Reference
	Yes	132 (48)	25 (81)	107 (44)	5.4 (2.1–13.6)
Any Antibiotics^c^	No	138 (50)	4 (13)	134 (55)	Reference
	Yes	138 (50)	27 (87)	111 (45)	8.1 (2.8–24.0)
Currently** on Allergy Medicines^	No	214 (78)	27 (87)	187 (76)	Reference
	Yes	62 (22)	4 (13)	58 (24)	0.5 (0.2–1.4)
Previous Ear Infections	No	95 (34)	1 (3)	94 (38)	Reference
	Yes	175 (63)	30 (97)	145 (59)	21.6 (2.9–160.9)
Adenoidectomy	No	253 (92)	30 (97)	223 (91)	Reference
	Yes	23 (8)	1 (3)	22 (9)	0.34 (0.04–2.6)
Previous Ear Tubes	No	195 (71)	25 (81)	170 (69)	Reference
	Yes	81 (29)	6 (19)	75 (31)	0.5 (0.2–1.4)
Cleft Palate	No	256 (93)	31 (100)	225 (92)	Reference
	Yes	20 (7)	0 (0)	20 (8)	—^#^

*Percent totals may not add up to 100% due to rounding error or lack of patient reporting

**At time of tympanostomy tube placement

^a^Current antibiotics include: Amoxicillin, Amoxicillin + Clavulanic Acid, Azithromycin, Cefdinir, Cefixime, Clindamycin, Sulfamethoxazole + Trimethoprim

^b^Previous antibiotics include: Amoxicillin, Amoxicillin + Clavulanic Acid, Ampicillin, Azithromycin, Cefdinir, Cefixime, Cefpodoxime, Cefprozil, Ceftibuten, Ceftriaxone, Cefuroxime, Ciprofloxacin + Dexamethasone, Clindamycin, Penicillin, Sulfamethoxazole + Trimethoprim

^c^Any antibiotics: Combination of results from patients currently or previously on antibiotics

^^^Current allergy medicines include: Budesonide, Cetirizine, Diphenhydramine, Fexofenadine, Fluticasone, Loratadine + Pseudoephedrine, Montelukast, Prednisone

^#^Odds ratio cannot be computed

Overall, 149 (54%) of 276 children had middle ear fluid that were PCR positive for the presence of *H*. *influenzae*, *S*. *pneumoniae*, *A*. *otitidis*, and/or *M*. *catarrhalis*. Of these 149 PCR positive samples, 117 (79%) identified a single organism and 32 (21%) were polymicrobial (Fig 1).

The three classic otopathogens were more likely to be identified as either a single organism or a polymicrobial component in purulent than non-purulent effusions (Tables 2 and 3). This pattern was observed for *H*. *influenzae* (52% vs. 18%, p<0.001), *S*. *pneumoniae* (19% vs. 4%, p<0.001) and *M*. *catarrhalis* (26% vs. 12%, p = 0.04) but not for *A*. *otitidis* (23% vs. 26%, p = 0.71).

**Table 2:** Bacterial DNA Identified in Middle Ear Fluid Based on Effusion Type.

		Purulent Effusions (n = 31)	Non-purulent Effusions (n = 245)	
		No. of Children (Column %*)	No. of Children (Column %*)	p-value
*Haemophilus influenzae*	Single Organism	10 (32)	30 (12)	<0.001
	Polymicrobial Component	6 (19)	15 (6)	
	None	15 (48)	200 (82)	
*Streptococcus pneumoniae*	Single Organism	3 (10)	7 (3)	0.002
	Polymicrobial Component	3 (10)	2 (1)	
	None	25 (81)	236 (96)	
*Alloiococcus otitidis*	Single Organism	2 (6)	46 (19)	0.07
	Polymicrobial Component	5 (16)	17 (7)	
	None	24 (77)	182 (74)	
*Moraxella catarrhalis*	Single Organism	4 (13)	15 (6)	0.07
	Polymicrobial Component	4 (13)	15 (6)	
	None	23 (74)	215 (88)	
Overall	Single Organism	19 (61)	98 (40)	<0.001
	Polymicrobial Component	8 (26)	24 (10)	
	None	4 (13)	123 (50)	

*Percent totals may not add up to 100% due to rounding error

**Table 3:** Comparison of Purulent and Non-purulent Bacterial DNA Prevalence.

	Purulent Effusions (P)	Non-purulent Effusions (N-P)	
	No. of Patients (%) (n = 31)	No. of Patients (%) (n = 245)	Fold Difference (P vs. N-P)
*Haemophilus influenzae*	16 (52)	45 (18)	2.9
*Streptococcus pneumoniae*	6 (19)	9 (4)	4.75
*Moraxella catarrhalis*	8 (26)	30 (12)	2.2
*Alloiococcus otitidis*	7 (23)	63 (26)	0.88

A single organism was identified in 61% percent (19 of 31) of all purulent effusions (Table 2), most frequently *H*. *influenzae* followed by *M*. *catarrhalis*. *S*. *pneumoniae* and *A*. *otitidis* were identified as single organisms in a minority of purulent effusions. All four otopathogens contributed to the 26% of polymicrobial purulent effusions.

A single organism was identified in 40% (98 of 245) of non-purulent effusions (Table 2). *A*. *otitidis* accounted for almost half the single organisms identified and *H*. *influenzae* accounted for about a third, with *S*. *pneumoniae* and *M*. *catarrhalis* being identified less frequently. Overall, 10% of non-purulent effusions were polymicrobial, containing *H*. *influenzae*, *A*. *otitidis*, and *M*. *catarrhalis* with similar frequency and infrequently harboring *S*. *pneumoniae*.

*H*. *influenzae* was identified 2.9x more frequently in purulent effusions than non-purulent effusions (52% vs. 18%); *S*. *pneumoniae* was identified 4.8x more frequently in purulent effusions than non-purulent effusions (19% vs. 4%); *M*. *catarrhalis* was identified 2.2x more frequently in purulent effusions than non-purulent effusions (26% vs. 12%). In contrast, *A*. *otitidis* was present in relatively equal amounts in purulent effusions and non-purulent effusions (23% vs. 26%) (Table 3) In adjusted and unadjusted multiple logistic regression analyses (Table 4), predictors of purulent as opposed to non-purulent middle ear fluid were *H*. *influenzae*, *S*. *pneumoniae*, and age <1 year.

**Table 4:** Factors Associated with Purulent as Opposed to Non-purulent Middle Ear Effusions.

Variable		Unadjusted Odds Ratio (95% Confidence Interval)	Adjusted Odds Ratio (95% Confidence Interval)
Organism	*Haemophilus influenzae*	4.7 (2.2–10.3)	4.3 (1.8–10.1)
	*Streptococcus pneumoniae*	6.3 (2.1–19.1)	5.6 (1.5–20.2)
	*Alloiococcus otitidis*	0.8 (0.3–2.1)	1.1 (0.4–2.9)
	*Moraxella catarrhalis*	2.49 (1.02–6.07)	2.0 (0.8–5.6)
Age	<1 year	Reference	Reference
	1–3 years	0.5 (0.2–1.3)	0.8 (0.3–2.2)
	>3 years	0.09 (0.02–0.35)	0.20 (0.04–0.90)

## Discussion

This prospective study evaluated the presence of four otopathogens (*H*. *influenzae*, *S*. *pneumoniae*, *A*. *otitidis*, and *M*. *catarrhalis*) in middle ear fluids obtained from children undergoing routine tympanostomy tube placement at Wake Forest Baptist Medical Center from January 2011 through March 2014. Our major findings were: (1) analyses of purulent effusions most often yielded the identification of a single bacterial organism rather than a polymicrobial mixture; (2) the most prevalent single organism identified in purulent effusions was *H*. *influenzae*; (3) ~90% of purulent effusions indicated the presence of one or more of the otopathogens; (4) *A*. *otitidis* was the most common organism identified in non-purulent effusions; (5) half of the non-purulent effusion samples indicated the absence of all of the otopathogens being evaluated; (6) the overall prevalence of *S*. *pneumoniae* in effusion samples was low; (7) *H*. *influenzae*, *S*. *pneumoniae*, and *M*. *catarrhalis* all occurred ≥2-fold more prevalently in purulent effusions than non-purulent effusions; however, *A*. *otitidis* occurred equally among purulent and non-purulent effusions.

Our results obtained with purulent effusions correlate with a potentially developing trend in AOM etiology. Historically, the bacterial organisms most commonly identified as AOM pathogens have been *S*. *pneumoniae*, *H*. *influenzae*, and *M*. *catarrhalis*[16–19]. After introduction of the heptavalent pneumococcal conjugate vaccine PCV7 in 2000, *H*. *influenzae* briefly became the most prevalent otopathogen identified in cases of OM[20, 21]. Shortly thereafter, serotypes of *S*. *pneumoniae* not covered in the initial conjugate vaccine design became prevalent in OM cases and were identified in proportions equaling that of *H*. *influenzae*[22–24]. In 2010, a new vaccine incorporating PCV7 *S*. *pneumoniae* serotypes as well as 6 new emerging serotypes was released for widespread usage in the United States. Recent data based on results from Rochester, NY detailing the prevalence of bacteria causing AOM in North America in 2012 suggest that a shift in OM bacterial etiology may again be occurring[25]. Briefly, *H*. *influenzae* has reemerged as the predominant AOM pathogen, followed by *M*. *catarrhalis*. The prevalence of *S*. *pneumoniae* in cases of AOM has decreased to levels below those of the other two common otopathogens. The purulent effusion data obtained in our study revealing high levels of *H*. *influenzae* and *M*. *catarrhalis* in conjunction with low levels of *S*. *pneumoniae* are similar to the results described by Casey and Pichichero[25] and Zhao et al[26]. Notably, our subject population underwent tympanostomy tube placement for clinical indications so that most would have recurrent or chronic OM infection and may differ from studies of children from outpatient pediatric clinics, which could include first episodes of otitis media. Also, our designation of purulence based on visual subjectivity rather than molecular quantification represents a potential limitation to this study.

*A*. *otitidis* is a slow-growing organism that has been historically overlooked as an OM pathogen due to the use of standard bacterial culture techniques when evaluating organisms present in OM middle ear fluid. Because *A*. *otitidis* grows poorly in standard culture, the increasing use of PCR to identify the etiology of otitis media has resulted in an increased recognition of its presence in middle ear fluid. Our finding that *A*. *otitidis* is the most prevalent organism in non-purulent effusions is consistent with what has been seen in other recent literature[14, 27, 28].

As was seen in our purulent effusions, *S*. *pneumoniae* was also the least identified organism in the non-purulent effusion samples. The overall low prevalence of *S*. *pneumoniae* in middle ear fluid in our study is consistent with what is being reported in current OM literature [14, 25, 26, 28–30]. Taken together, our results and current literature may suggest that the pneumococcal conjugate vaccines have significantly reduced the ability of *S*. *pneumoniae* to cause recurrent OM.

Another interesting finding lies in the different frequency of identifying each organism when purulent effusions are compared with non-purulent effusions. *H*. *influenzae*, *S*. *pneumoniae*, and *M*. *catarrhalis* were detected ≥2.2x more frequently in purulent effusions than non-purulent effusions. Because OME can persist for several months after AOM[25], decreased frequency of identification of these pathogens from non-purulent effusions may be partly explained by bacterial clearance following antimicrobial treatment and/or clearance by human immune defense mechanisms.

Although the above differences were apparent for the 3 most common otopathogens, *A*. *otitidis* results follow a distinct pattern. The fact that *A*. *otitidis* percentages remained stable in cases of purulent and non-purulent effusions may suggest that the organism is a commensal. Another possible explanation is that *A*. *otitidis* is able to more effectively persist in the middle ear than the three classic otopathogens. According to the literature, strains of *A*. *otitidis* lack β-lactamase and can exhibit intermediate levels of resistance to β-lactam antimicrobials as well as cephalosporins[31]. Given that current OM treatment guidelines recommend amoxicillin (a β-lactam) as the first drug of choice, the resistance observed in *A*. *otitidis* would decrease the efficacy of this drug. Given that *A*. *otitidis* contains no β-lactamase, a combination of amoxicillin and clavulanate would also be less effective against this organism than the classic otopathogens. One could speculate that the described resistances to common OM antimicrobial treatments could facilitate middle ear persistence.

## Conclusions

The work presented here describes the prevalence of 4 bacterial otopathogens in middle ear fluid obtained from children undergoing tympanostomy tube placement. Our results suggest that *H*. *influenzae* has surpassed *S*. *pneumoniae* as the most common pathogen identified in cases of purulent effusions, a result that may have possible implications in antimicrobial treatment guidelines. Although *S*. *pneumoniae* was infrequently identified, the odds of a purulent versus non-purulent infection were significantly associated with identification of *H*. *influenzae*, *S*. *pneumoniae*, and with age <1 year.
